# Lost Food and Associated Phosphorus Footprint: Evidence from China

**DOI:** 10.3390/foods13081262

**Published:** 2024-04-20

**Authors:** Dengyun Gao, Xing Li, Junkai Ma, Long Qian

**Affiliations:** 1 Institute of Food and Strategic Reserves, Nanjing University of Finance and Economics, Nanjing 210023, China; gaody900106@163.com (D.G.); majunkai0427@163.com (J.M.); 2 School of Economics and Management, Taizhou University, Taizhou 225300, China; 3 School of International Economics and Trade, Nanjing University of Finance and Economics, Nanjing 210023, China; lixing@nufe.edu.cn

**Keywords:** phosphorus footprint, food waste, Chinese university canteens, food security, environmental impact

## Abstract

The environmental impacts of excessive phosphorus emissions (PE) have been widely discussed in recent years. This study aims to calculate and evaluate the phosphorus footprint (PF) of food thrown away in Chinese universities. Based on a nationwide survey involving 9192 university students from 29 provinces and 29 universities in China, the result reveals that the PF generated by food waste in Chinese university canteens was 3.209 Kt in 2018. Furthermore, it is found that meal satisfaction, gender, regional economic level, dietary culture, and years of education all have significant impacts on lost food PF. Our findings emphasize the importance of reducing food waste in university canteens, which plays a crucial role in ensuring food security and environmental protection.

## 1. Introduction

There is a widespread concern regarding food waste and loss among policymakers and the academic community [1,2,3]. Steinfeld [4] and Kummu et al. [5] indicate that a third of the world’s food is lost or wasted during different stages of the food supply chain. The United Nations recently set a goal to halve the per capita global food waste by 2050. There is a widespread belief that developing countries suffer more food loss during the initial phases of the food supply chain, such as production and post-harvest handling, due to a lack of adequate financial, technical, and managerial resources. Conversely, food waste during the consumption stage is generally lower than in developed countries [5,6]. However, with the economic development and change in dietary patterns in developing countries, this pattern may change [7]. With the development of the Chinese economy, food waste at the consumption stage in China is becoming more and more common [8]. Some studies have found that Chinese consumers’ food waste at home and outside the home, including in public canteens and restaurants, is becoming increasingly common and cannot be underestimated [8,9]. With a population of more than 1.4 billion, China could play an important role in the global effort to reduce food waste [10], thus it is necessary to strengthen China’s research in this area.

Phosphorus fertilizer is a major component supporting the ecosystem’s material cycles [11], but excessive phosphorus emissions are becoming a serious global challenge [12] and have resulted in environmental problems such as water pollution, eutrophication, and hypoxia [13,14]. Thus appropriate actions should be taken to achieve food security and environmental improvement [15]. How to prevent excessive phosphorus emissions has become a hot topic among the academic community. Dumas et al. [16] modeled the biogeochemical processes of phosphorus in the global food supply and identified two critical relationships affecting crop yields. Neset [17] investigated the phosphorus production–consumption flow mechanism in a Swedish city and discovered that the main reason for the growth in overall per capita phosphorus emissions is the consumption of livestock products, which are also a source of phosphorus. 

Food waste not only brings huge economic losses and threatens food security, but also brings about a series of negative environmental effects, such as excessive phosphorus emissions. Therefore, reducing phosphorus emissions could be achieved by reducing food waste. The literature that is closely related to our study discusses measuring the PF of lost food. Alnadari et al. [18] studied food products’ PF in Yemen in the past 57 years. Based on a revised model of the N-Calculator, the results show that the PF (Gg P year^-1^) in Yemen developed from 6.77 to 122 over the last 57 years. Although there is an increasing number of studies assessing lost food PF [19,20], policymakers are hindered by the lack of reliable data regarding the extent of food waste [21]. The prevalent problems observed in academic literature assessments on food waste include deficiencies in data [22] and excessive dependence on secondary data [13] instead of the direct assessment of lost food parameters [23].

Chinese university canteens are important sources of food waste outside the households [24]. For a developing country such as China, where both the number of students enrolled and the number of universities are among the highest in the world, studying China’s food waste in universities is urgent. However, to the best of our knowledge, almost all research pertaining to food waste in universities has been carried out in developed countries; for instance, a study on food waste among 205 students was performed at Rhodes University in South Africa [25]; a tracking survey on canteen food waste was undertaken at the Lisbon University College of Agriculture in Portugal [26]; and a case study was performed at Ghent University in Belgium [27]. 

This study aims to assess the lost food PF in Chinese university canteens, and to further explore its key influencing factors. It is important because food waste not only leads to massive resource waste [5], but also the nutrients in food are lost, as well as phosphorus and nitrogen being discharged into the water body, which could seriously interfere with the water purification process, thus causing a great number of deaths of aquatic organisms [28]. Consequently, assessing and taking effective measures to reduce food waste and the corresponding PF are crucial for maintaining food security and protecting the environment. As a “major disaster area” of food waste other than households and restaurants [29], universities have a responsibility and obligation to take positive actions to decrease lost food PF. Moreover, it is necessary for all stakeholders to understand the key influencing factors of lost food PF, which will help us to develop more effective strategies to reduce lost food PF. Therefore, studying food waste and the corresponding PF is necessary and urgent, especially in an emerging country such as China, which has the largest number of university students in the world.

The marginal contributions are as follows. Firstly, compared to small-scale surveys documented in the existing literature, we provide a more reliable estimation of the PF of lost food in Chinese universities based on a nationwide survey. Secondly, the PF of lost food in university canteens, as an important place for food waste, is measured here for the first time in China. This helps us understand the environmental impacts resulting from Chinese university canteens’ food waste. Thirdly, we explored the determinants of lost food PF in Chinese university canteens and concentrated on making a comparative analysis from the perspectives of individual characteristics, dining characteristics, regional characteristics, and dietary cultural characteristics. This helps us to better understand the PF of lost food produced by different individuals.

## 2. Materials and Methods

### 2.1. Data Collection

**Figure 1:**
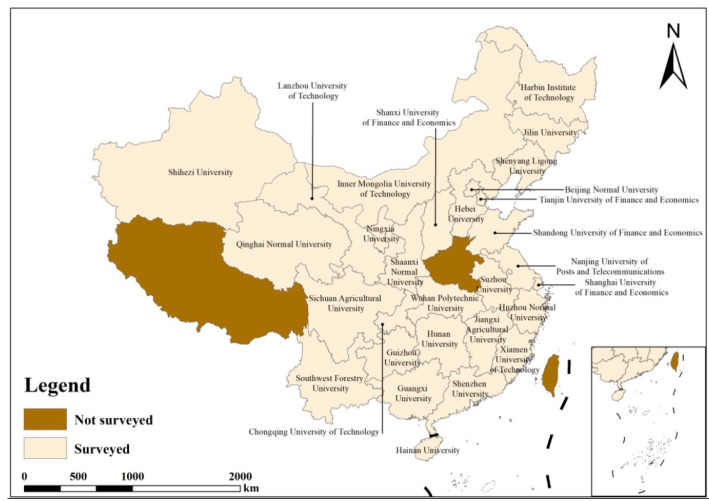
Sample distribution.

In 2018, a national survey was carried out on the subject of “Lost food in Chinese university canteens”. Following the principle of “one university surveyed in one province”, one university was randomly chosen in each of the 30 provinces in China (excluding Tibet, Hong Kong, Macau, and Taiwan). A questionnaire survey was conducted in 30 universities—one university in each of the 30 provinces. Approximately 300–350 samples of canteen food waste were randomly selected from most of these universities. Regretfully, we do not have access to reliable data for Henan Province. As a result, 9192 valid questionnaires from 29 universities in 29 provinces were ultimately utilized for the canteen food waste research in this study. The universities surveyed are shown in Figure 1 below. 

### 2.2. The Survey Designs

There are two sections in the survey questionnaire. Questions about personal profiles, household characteristics, food-saving initiatives, dining facility services, and other information were included in the first section of the survey questionnaire. The purpose of the second section was to record the quantity and composition of food waste that each respondent at the university canteen generated. Table 1 presents the fundamental data obtained from the samples.

To acquire precise data, our team recruited and trained 10 investigators for each of the chosen universities to help them become familiar with the food waste weighing protocols and the questionnaire process. Additionally, one supervisor was appointed for each of the chosen universities to ensure the quality of the data collected in the survey. In each of the chosen university canteens, the investigators randomly selected university students who were dining in the canteen and began data collection after obtaining their consent. To avoid potential influence on the dining behavior of the university students, the surveys were only conducted after the students had finished their meals. Two trained investigators worked together, with one responsible for categorizing and weighing the leftover food on the plates. This investigator strictly classified and weighed the leftover food items on the plates according to the type of ingredients to obtain data on various types of food waste. The data were measured in grams and precise to two decimal places. The other investigator was responsible for recording the food waste data and obtaining information about each respondent’s individual, family, and socioeconomic status. Both the data collection from the questionnaire survey and the food waste collection process were conducted under the full supervision of the respective quality supervisors to ensure, as much as possible, the accuracy of the data collected. Finally, after nearly over two months, and the efforts of the 29 supervisors and 290 investigators, we accumulated more than 30,000 measurements and obtained food waste data from 9192 university students dining in each of the university canteens in the 29 chosen provinces in mainland China.

### 2.3. Food Waste Measurement

Two difficulties must be addressed in order to obtain accurate food waste data from university students dining in canteens. To begin with, a precise definition of food waste is essential. Food waste is defined in this study, based on the definition of the FAO, as a waste of edible food preventable under existing conditions [30,31]. Inedible portions (such as bones, eggshells, vegetable skins) are not considered as food waste. Furthermore, in comparison to similar studies conducted at Western institutions, food waste was confined to solid waste, with soup, cooking oil, beverages, and milk being excluded [32,33]. To be consistent with previous studies, the main types of food were classified into five major categories [9], including vegetables, meats (pork, poultry, bovine and mutton), aquatic products, eggs, and grains (rice, soy products, and wheat). Second is the selection of an appropriate approach to collect the data on food waste in university canteens. To get accurate and neutral data, this study used the direct weighing method. Although this method is more labor-intensive and time-consuming, it has the advantage of being able to obtain accurate data on food wastage [9].

### 2.4. Phosphorus Footprint Analysis

“Phosphorus footprint”, as a relatively new technology, is derived from the ecological footprint put forward by Rees and Wackernagel [34]. Up to now, there is no unified conclusion on the concept of PF in the academic field. Drawing on Matuštík et al. [35], lost food PF is defined as the overall quantity of phosphorus released into the environment resulting from food waste. Yet, existing studies on PF are lacking. Some scholars who studied PF mainly concentrated on the agricultural or food field, such as Bizimana et al. [36], who estimated the PF of Rwanda’s agricultural food system from 1961 to 2020; while Metson et al. [15] mainly focused on the impact of dietary choices on PF of an average Australian city; and Oita et al. [28] assessed and compared the trends of PF in Asia.

### 2.5. Calculation of Lost Food PF

Usually, food waste data are directly obtained by electronic scales [37]. However, this approach ignores the inevitable loss of moisture via cooking during the processing of raw food into cooked food. Thus, drawing on Wang et al. [38], in order to calculate the quantity of food waste more accurately, we used the conversion coefficient (C1k) to transform the amount of cooked food waste into the associated raw food quantity. In addition, energy and material dissipation also occur during the process of converting agricultural products into raw food, resulting in increased phosphorus emissions per unit of consumed product [39]. This study intends to further calculate lost food by using the conversion coefficient (C2k) between raw food and agricultural products [38,40]. Specific conversion coefficients and data sources are shown in Table 2. Drawing on the study of Wang et al. [38] and Qian et al. [9], the calculation formula of lost food PF after conversion is shown in Equations (1)–(5).

(1) $$W_{k}={FW}_{ik}\times C_{1k}\times C_{2k}\times 2$$

In Equation (1), ${FW}_{ik}$ is the food waste of specific food k that student i wastes per meal at the Chinese university canteen; $C_{1k}$ is the food category’s conversion coefficient from cooked to raw food; $C_{2k}$ is the conversion coefficient from raw food to agricultural products for the k category of food; $W_{k}$ is the amount of food waste in the k category after conversion (g/d). Considering the simplicity and low waste rate of breakfast served in the Chinese university canteens, this study measures the daily food waste per capita in the Chinese university canteens by usingstating two meals per day (including lunch and dinner only) as the accounting standard [24].

(2) $${FW}_{k}=\frac{\sum_{i=1}^{9192}W_{k}}{9192}$$

In Equation (2), the daily per capita food waste amount (g/d) for the k category of food at Chinese university canteens is represented by the symbol ${FW}_{k}$.

(3) $${FW}_{D}=\sum_{k=1}^{5}{FW}_{k}$$

In Equation (3), ${FW}_{D}$ represents the daily per capita food waste (g/d).

(4) $${FW}_{T}={FW}_{D}\times N\times 270$$

In Equation (4), N represents all Chinese students enrolled in universities in 2018, N ≈ 40 million; 270 is the number of days of dining in university canteens for university students (excluding winter and summer holidays); ${FW}_{T}$ represents the total food waste at the Chinese university canteens for the entire year (Kt).

(5) $${PF}_{D}={FW}_{D}\times P_{k}$$

In Equation (5), the converted daily per-person PF of lost food in Chinese university canteens is represented by the symbol ${PF}_{D}$(gP/d); $P_{k}$ is the amount of phosphorus in different agricultural products (gP/g), as shown in Table 3.

## 3. Results

### 3.1. Amount and Composition of Food Waste

**Figure 2:**
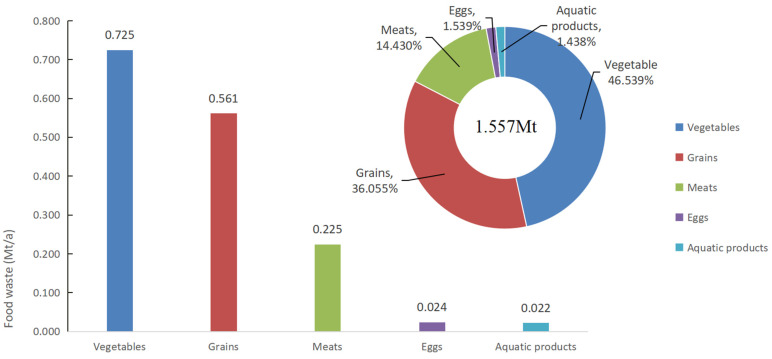
Amount and composition of food waste in Chinese university canteens.

The findings indicate that in 2018, Chinese university canteens’ per capita food waste was 144.176 g/d after conversion. With over 40 million university students in China in 2018 and an average of 270 days spent on campus annually, the total food waste in Chinese university canteens (${FW}_{T}$) can be calculated as 1.557 Mt. As for the composition of lost food, vegetable waste is the highest, at about 0.725 Mt, accounting for 46.539%, followed by grain, at 36.055% (0.561 Mt) and meats at 14.430% (0.225 Mt); only 1.539% (0.024 Mt) and 1.438% (0.022 Mt) of the total food waste is accounted for by eggs and aquatic products, respectively, as shown in Figure 2.

### 3.2. Size and Structure of Lost Food PF

**Figure 3:**
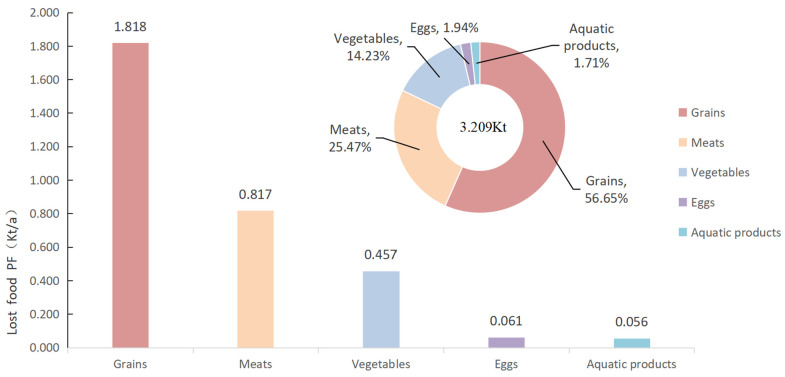
Size and structure of the phosphorus footprint (PF) of lost food at university canteens.

Our results indicate that in 2018, the lost food PF in Chinese university canteens amounted to 3.209 Kt. In terms of lost food structure, grains contain the highest PF, accounting for 56.65% (1.818 Kt) of the overall PF of lost food, followed by meats, with the PF accounting for 25.47% (0.817 Kt) of the total PF, and with pork containing the largest PF (0.651 Kt). The PF of vegetables is slightly lower than that of meats, accounting for 14.23% of the total PF. With 1.94% and 1.71% of the overall PF, respectively, eggs and aquatic products have relatively low PF, as shown in Figure 3.

### 3.3. Spatial Distribution of Lost Food PF

**Figure 4:**
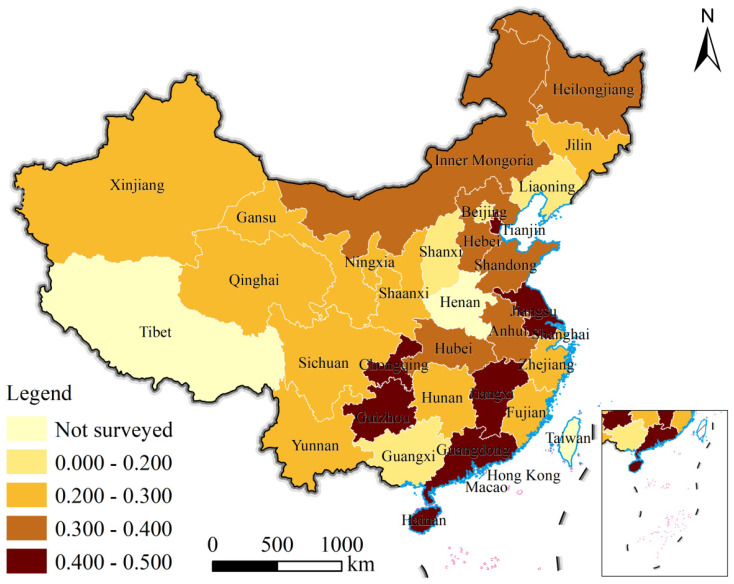
Spatial distribution of lost food PF.

There are significant regional differences in the lost food PF in Chinese university canteens (Figure 4). The findings reveal that the effects of regional economic levels on the PF of lost food are significant. For instance, the PF of lost food in the mid-west is significantly lower than that in the east. Moreover, staple food consumption patterns also affect lost food PF greatly [43,44]. For instance, the highest per capita PFs of lost food (0.477 gP/d and 0.449 gP/d, respectively) are found in the southern Chinese provinces of Jiangsu and Guangdong, which are primary rice-consuming regions. In contrast, the per capita lost food PF of the Liaoning and Beijing provinces (mainly wheat consumers) in northern China was less than 1/3 of that of Jiangsu (0.122 gP/d). Tianjin, Guangdong, Jiangsu, and Hainan had the four highest per capita lost food PFs in the country (0.495–0.446 gP/d), followed by the other places in the eastern and central regions of China. The northwest and northeast regions of China have the lowest PF. Thus, reducing the PF of lost food can be achieved more effectively by altering the waste behavior of university students who consume rice in economically developed regions. 

### 3.4. Relationship between PF and Some Key Features 

**Figure 5:**
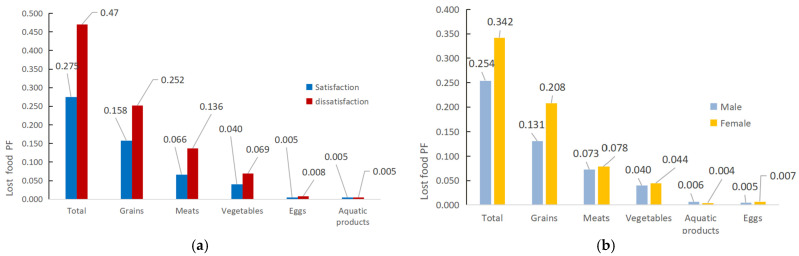
Meal satisfaction, gender, and lost food PF (gP/d). (**a**) Meal satisfaction and lost food PF. (**b**) Gender and lost food PF.

Previous studies have shown that the individual characteristics of consumers, catering characteristics, regional characteristics and dietary culture are closely related to food waste [9]. This also means that these factors and the PF may also be closely related. Taking into account the questionnaire protocol and data acquisition, in this section, we mainly discuss the relationship between PF and gender, years of education, meal satisfaction, dietary culture, and regional economic level [24]. The correlation between meal satisfaction and lost food PF is depicted in Figure 5a. The findings show that university students’ meal satisfaction has a significant impact on their PF of lost food, as evidenced by the fact that students satisfied with their meals have a PF of 0.275 gP/d, which is significantly lower than students dissatisfied with their meals (0.47 gP/d). Regarding waste types, grains, meats, and vegetables show a greater drop in PF with increased meal satisfaction compared to eggs and aquatic products. Thus, in line with Lorenz’s findings [45], meal satisfaction can contribute to a decrease in food waste and consequently the PF of Chinese university canteens.

The relationship between gender and lost food PF is displayed in Figure 5b. We found that lost food PF in the female group (0.342 gP/d) in Chinese university canteens is higher than in the male group (0.254 gP/d). The results illustrate that gender is an important influence factor of the PF of lost food. The main reason is the significant difference in the PF of grain waste between the two groups, whereby female students show significantly higher levels of staple food waste than male students. The conclusion is consistent with the findings of Wu et al. [10]; that is, gender significantly negatively influences staple food waste, indicating that male students produce a lower level of staple food waste than female students. Therefore, in order to satisfy the demands of female students and lower food waste and the associated PF, university canteens should be encouraged to offer a smaller portion of staple foods.

**Figure 6:**
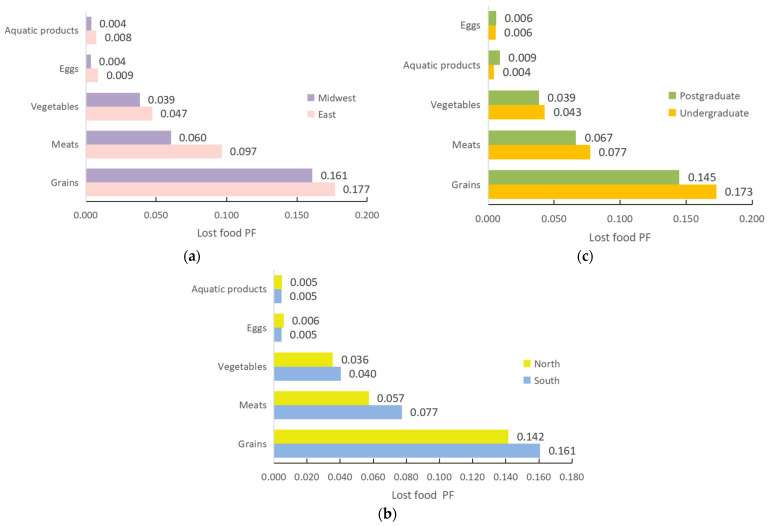
Other important influencing factors and lost food PF (gP/d). (**a**) Regional economic level and lost food PF. (**b**) Dietary culture and lost food PF. (**c**) Education and lost food PF.

Furthermore, there are other factors affecting the university canteens’ lost food PF. Firstly, Figure 6a shows the per capita lost food PF of university students in eastern China is 0.336 gP/d, while in mid-western, China it is only 0.267 gP/d (after calculation), showing the phenomenon of high per capita lost food PF in the east and low values in the west. The primary cause is that meat greatly affects the differences in lost food PF between the east and the mid-west. The per capita PF of lost meat in the east is significantly higher than that in the mid-west, reaching 0.037 gP/d. The difference in the waste PF of other food categories is not obvious. The finding is consistent with the conclusion of Bilgic et al. [46], who found that expenditure elasticity for bovine and mutton is significantly positive at the 1% level and the expenditure elasticity value is 1.428, indicating that the economic level and household income can increase the consumption and waste of meat products. Therefore, the regional economic level is an important influencing factor of lost food PF. 

Secondly, dietary culture is also an influential factor in the lost food PF of university canteens (Figure 6b). Drawing on Talhelm et al. [44] and Qian et al. [24], southern China is a traditional rice-growing area, while the north is mainly a wheat-growing region, which leads to significant dietary culture differences between the south and the north. The per capita PF of lost food among university students in the south is 0.287 gP/d, and in the north it is 0.245 gP/d (after calculation), with a more pronounced trend of high in the south and low in the north. In terms of the structure of lost food PF, the difference between per capita meat waste PF and per capita grain waste PF is the largest, with values of 0.020 gP/d and 0.019 gP/d, respectively, followed by vegetables, with a difference of 0.004 gP/d between the north and the south, while the difference between eggs and aquatic products is smaller. This means that the difference in the PF of lost food between the north and the south in university canteens is mainly caused by the divergence of food waste performance between staple food and meats, which fading is consistent with that of the research by Qian et al. [24], who found that food waste in “northern China” was statistically significant, indicating that university students in northern China generated less food waste than those in southern China. 

Finally, the lost food PF of undergraduate students is significantly higher than that of postgraduate students (Figure 6c). In particular, the per capita lost food PF of undergraduate students is 0.303 gP/d, while the same indicator for postgraduate students is only 0.265 gP/d (after calculation). This finding confirms the conclusion of Florkowski et al. [47], indicating that years of education can decrease food waste and loss. Therefore, an increase in years of education can help reduce food waste and the corresponding PF.

## 4. Discussion

### 4.1. Lost Food and the Embedded PF

We further performed a comparative analysis of food waste (Table 4). Firstly, we calculated that the food waste mass per student per meal is about 72 g based on data from 29 universities in mainland China. This is less than the food waste mass per student per meal of university students in developed regions, as demonstrated by Wu et al. [10] in six Beijing universities and Zhang et al. [48] in seven Wuhan universities. One possible explanation for this could be that food waste in Beijing and Wuhan is higher due to the developed economic level; one of the two cities is the capital of China and the other is a new first-tier city in the central region. However, our findings are based on a national survey, making the findings more representative at the national level. Secondly, we found that university canteens’ food waste is lower compared to food waste generated in restaurants, based on the study of Wang et al. [31], Wang et al. [38], and Xu et al. [37]. Thirdly, we also found that food waste in university canteens is higher compared to household food waste, based on the study of Min et al. [49] and Li et al. [50]. Lastly, according to the studies by Painter et al. [25], Pinto et al. [26], and Ellison et al. [51], it is not difficult to conclude that Chinese university students generate lower food waste than those in Western countries. The possible reason is that the more developed the economic level is, the higher the food waste is. Due to the high correlation between food waste and the generated phosphorus footprint, it is not difficult to draw the following conclusions from the comparison: at the per capita level, the negative impact of individual food waste on the environment when eating in university canteens is greater than that of family places and lower than that of restaurants, and compared with Western universities, the negative environmental effect of food waste in Chinese universities is smaller.

Secondly, the determining factors affecting lost food and corresponding PF were revealed. This implies that several blended strategies could serve as the foundation for lost food reduction efforts. We discovered that factors such as gender, years of education, meal satisfaction, dietary culture, etc., were associated with each measure’s efficacy. Therefore, targeted policies should be adopted based on different consumer characteristics.

Thirdly, the study also emphasized that the emission of phosphorus from food waste can cause serious environmental pollution. The overall PF of lost food at the Chinese university canteens was found to be 3.209 Kt. The environmental impact of lost food PF mainly includes two parts: soil accumulation and water pollution. Therefore, food waste not only brings about hidden dangers to food security, but also causes serious environmental pollution problems. 

### 4.2. Potential Mitigation Strategies

As a “major disaster area” for food waste, other than households and restaurants, university canteen management should attach great importance to the canteens’ food waste and the waste behavior of university students. We found that it is a common phenomenon for university students to waste food. Thus, lost food and the corresponding PF should not be underestimated. However, there is currently a serious lack of interest in food waste and the environmental impact of Chinese university canteens. Universities and canteen management should strive to reduce food waste and environmental pollution. Consequently, this study could contribute to the implementation of policies to reduce food waste. 

Firstly, continuously improving the quality of canteen meals and enhancing university students’ meal satisfaction could help in reducing lost food and the corresponding PF. Therefore, university canteens should strengthen communication with students, strive to improve the taste of dishes, continuously innovate and improve dishes, establish a high-quality and diverse food supply system, and try to meet the daily dietary needs of different groups as much as possible, thereby curbing food waste at the root. 

Secondly, we should focus on the food waste of specific groups, such as female and undergraduate students. The lost food PF of female students and undergraduates is relatively high, and it is recommended to further explore the deep-seated reasons, such as the differences in dietary consumption, consumption concepts, and environmental awareness of different groups. Thus, targeted lost food reduction measures could be formulated, such as encouraging university canteens to provide smaller portions, combinations of dishes, and other food supply systems to facilitate university students to purchase meals according to their needs. In addition, there is a need to strengthen the promotion of and education around the prevention of food waste among undergraduate students, thereby enhancing their environmental awareness and reducing food waste. 

Thirdly, the more developed the regional economy is, the higher the lost food PF among university students will be. It can be foreseen that with the continuous growth of the Chinese economy and the increase in the consumption of high-PF foods such as meat, lost food and associated PF at Chinese university canteens could increase further. Therefore, on the one hand, while maintaining the nutritional balance, university students should be urged to improve their dietary structure and avoid the excessive pursuit of high-PF foods; on the other hand, the government should introduce relevant legal measures to guide university students to minimize food loss and the ecological pressure caused by it. Fortunately, in 2021, the government enacted the “Anti Food Waste Law”, emphasizing that the whole of society should cherish food and reduce food waste. Of course, due to this only recently having been introduced, the actual effect remains to be observed. 

Lastly, there is a need to pay attention to the impact of dietary culture on lost food and the corresponding PF. Our study illustrates that the differences in lost food PF between the southern and northern regions are due to staple food and meat waste, which is closely associated with the dietary culture of the respective regions. Therefore, regional dietary culture is closely associated with lost food and corresponding PF. 

### 4.3. Limitation

Firstly, while this study highlights the possible influencing factors affecting the PF of lost food, other influencing factors remain unexplored, such as respondents’ subjective attitudes, as well as canteen dining environment (such as overcrowding and cleanliness). Furthermore, the fact that respondents had to respond to questions posed by our skilled investigators may also have made some of them reluctant to offer accurate information out of concern for their privacy, which could have diminished the reliability of the findings of our study. Secondly, our study only focused on university students and did not consider the characteristics of food waste among other groups in university settings, which can also lead to an underestimation of the lost food and the corresponding PF. Thirdly, with the rise of the delivery industry, the delivery of food for consumption has gradually become an alternative consumption choice for Chinese university students. However, this study did not solicit data pertaining to the delivery of food services, and therefore, since this paper did not examine the impact of food delivery services on food waste, further research is needed in this area in the future.

## 5. Conclusions

Food waste in university canteens and its environmental effects should not be ignored, especially given the excessive phosphorus emissions from food waste. Based on a nationwide survey of 29 university canteens in 29 provinces of China, this paper evaluated the food waste and corresponding PF of Chinese university canteens for the first time. It has been found that: (1) Food waste at Chinese university canteens was 1.557 Mt in 2018, with about 72 g of food waste per person per meal, which is lower than that at restaurants and higher than in households. Moreover, in terms of per capita waste, Chinese universities’ food waste is less than that in Western universities. (2) Chinese university canteens’ lost food PF was 3.209 Kt in 2018; lost grain PF was the highest, reaching 1.818 Kt, accounting for 56.65% of the total lost food PF. This suggests that the PF of lost food cannot be ignored. (3) Meal satisfaction, gender, education, dietary culture, and regional economic level were closely related to the generation of lost food PF. Therefore, targeted policies should be adopted for different consumer groups. 

## Figures and Tables

**Table 1:** Fundamental data about the samples.

Features	Types	Valid Samples	Proportions
Gender	Female	4418	48.06%
	Male	4774	51.94%
Household income (CNY)	<3000	1029	11.19%
	3001–5000	2895	31.50%
	5001–10,000	3547	38.59%
	10,001–20,000	1308	14.23%
	>20,000	413	4.49%
Education status	Undergraduate	7466	81.22%
	Postgraduate	1726	18.78%
Meal satisfaction	Very Satisfied	120	1.30%
	Satisfied	439	4.78%
	Neutral	4098	44.59%
	Dissatisfied	4535	49.33%
Food-saving campaigns	Yes	6801	73.99%
	No	2391	26.01%
Survey days	Working days	6378	69.38%
	Weekend	2814	30.62%
Region	East	3771	41.02%
	Midwest	5421	58.98%

**Table 2:** Food conversion coefficient (unit: g/g).

Cooked Food	Conversion to Raw Food	C1k *	Conversion to Agricultural Products	C2k *
Vegetables	Vegetables	1.050	Vegetables	1.500
Pork	Pork	1.330	Pigs	1.630
Poultry	Poultry	1.370	Poultry	1.490
Bovine and mutton	Bovine and mutton	1.410	Cattle and sheep	2.180
Aquatic products	Aquatic products	1.100	Aquatic products	1.180
Eggs	Eggs	1.050	Eggs	1.180
Rice	Rice	0.450	Paddy	1.480
Soy products Wheat	Wheat and other grains	0.530	Wheat and other grains	1.490

* Based on Adelodun et al. [40] and Wang et al. [38], C1k and C2k are, respectively, adopted in this study. Since there is no conversion coefficient for soy products in the pertinent research, we approximately use the wheat conversion coefficient from Adelodun et al. [40]. The mean value of the bovine conversion coefficients is used in place of that for mutton because there is no mutton waste in the university canteens.

**Table 3:** The phosphorus amount in different agricultural products (g P/g) [41,42].

Food Category		$\mathrm{Phosphorus}\;\mathrm{Amount}\;(P_{k}$)
Vegetables	Vegetables	0.00063
Meats	Pork	0.00560
	Poultry	0.00150
	Bovine and mutton	0.00210
Aquatic products	Aquatic products	0.00256
Eggs	Eggs	0.00260
Grains	Rice	0.00254
	Soy products	0.00605
	Wheat	0.00381

**Table 4:** A comparison of food waste mass among existing studies.

Country	Location	Year	Food Waste Mass (g/cap/meal)	Source
China	Universities in 29 provinces	2018	After conversion: 72.09 Before conversion: 61.03	Our source
China	University in Beijing	2018	Before conversion: 73.70	Wu et al. [10]
China	University in Wuhan	2019	Before conversion: 135	Zhang et al. [48]
China	Restaurants in four Chinese cities	2015	After conversion: 93	Wang et al. [31]
China	Restaurants in Lhasa	2015	After conversion: 98	Wang et al. [38]
China	Restaurants in Beijing	2015	Before conversion: 172.30	Xu et al. [37]
China	Households in China	2004, 2006 and 2009	Before conversion: 42.560	Min et al. [49]
China	Households in Rural China	2015	Before conversion: 8.74	Li et al. [50]
South Africa	Rhodes University	2015	Before conversion: 555	Painter et al. [25]
Portugal	Lisbon University	2017	Before conversion: 458	Pinto et al. [26]
United States	A university in the mid-west	2016	Before conversion: 88.230	Ellison et al. [51]

## Data Availability

The original contributions presented in the study are included in the article, further inquiries can be directed to the corresponding author.
